# Effects of Salt Stress on Earthworm Function and Compost Quality During Vermicomposting of Kitchen Wastes

**DOI:** 10.3390/bioengineering13010038

**Published:** 2025-12-29

**Authors:** Hailiang Mao, Jungang Ding, Wenqi Huang, Kui Huang, Rongchuan Yang

**Affiliations:** 1 School of Environmental and Municipal Engineering, Lanzhou Jiaotong University, Lanzhou 730070, China; 2 College of Forestry, Gansu Forestry Voctech University, Tianshui 741000, China

**Keywords:** earthworm, humification, biodegradation, organic waste, vermicomposting

## Abstract

The high salt concentration in kitchen waste (KW) can impede the performance of subsequent biological treatment. However, the impact of salt stress on the quality of vermicomposting products generated from KW remains unclear. In this study, the effects of high salt concentration in KW on earthworm function and vermicompost quality were investigated by comparing two groups: a 1.5% salt (ST) group and a control (CK) group without salt. Results showed a significant decrease in the number and weight of earthworms in the ST (*p* < 0.01), with a mortality rate of 24.33% (*p* < 0.05) after vermicomposting. Compared to the CK, ST treatment resulted in a significant increase in catalase activity and a significant decrease in superoxide dismutase activity (*p* < 0.01). In addition, mucus secretion by earthworms decreased by 82.6% in ST (*p* < 0.01). Moreover, salt stress reduced KW humification during vermicomposting, lowering the humification index and β:α index by 23.7% and 41.2%, respectively. Microbial composition shifted under spatially heterogeneous selection pressures, leading to a 37.5% decrease in Ascomycota abundance, a 58.3% increase in Bacteroidetes abundance, and a 72.3% reduction in *Proteobacteria* abundance. Furthermore, the vertical stratification of physicochemical conditions significantly affected both microbial abundance and earthworm biomass in the ST treatment (*p* < 0.01), suggesting a salt–microbe–earthworm interaction mechanism. This study reveals that salt stress disrupts humification by impairing key microbial functions and ecological roles of earthworms during vermicomposting of KW.

## 1. Introduction

Due to rapid urbanization and rising living standards, kitchen waste (KW) generation has continuously increased, creating significant challenges for municipal solid waste (MSW) management. KW accounts for approximately 52.8–65.3% of total MSW [1] and has high organic content and high perishability. Without proper treatment, KW can cause serious environmental contamination [2]. Conversely, KW is a valuable resource rich in organic matter and nutrients. Appropriate treatment techniques can convert KW into useful products, thereby achieving reduction and recycling of solid waste. Currently, the primary treatment methods for KW include incineration, landfilling, composting, and anaerobic digestion. However, these approaches have notable limitations, and they fail to achieve low-carbon and sustainable KW treatment [3].

Vermicomposting is a low-cost and sustainable biological treatment method that utilizes earthworm biodegradation to transform organic waste into nutrient-rich vermicompost [4]. Many KW materials have been proven to be usable for vermicomposting, like vegetables, fruits, rice, etc. [5,6]. The final vermicompost products of KW are rich in diverse probiotic groups, which can promote soil microbial activity and crop growth [7]. In addition, the leachate generated during KW vermicomposting can be transformed into earthworm tea, a valuable liquid fertilizer [8]. However, the composition of KW is highly complex, and local dietary habits can influence its compositional characteristics, including salt content. For instance, Chinese-style food waste typically contains approximately 2% salt [5]. Such salt accumulation, which varies across regions and cuisines, has been shown to negatively affect biological treatment processes. The latest research shows that high salinity conditions impose osmotic stress on earthworms and restrict their locomotor activity, thereby delaying the onset of vermicomposting [6,9]. Concurrently, elevated salinity markedly suppresses microbial proliferation, disrupts community structure and assembly mechanisms, and diminishes overall biodegradation capacity [6]. Therefore, in practical applications, the high salt content of KW has become a crucial factor restricting the efficiency and stability of the vermicomposting process.

Salt stress can impair the physiology, reproduction, and survival of earthworms through toxicological research. For example, Fang et al. [10] revealed the significant toxic effects of NaCl on earthworms, with the LC50 values of 4.1 g/kg and 3.6 g/kg on the 14th and 28th days, respectively, while earthworm mortality increased with increasing salt concentrations. Similarly, Raiesi et al. [11] found that, at high salinity levels (>4 g/kg), fresh weight of earthworms, cocoon production, and total number of earthworms decreased by 22%, 100%, and 53%, respectively, with severe tissue oxidative damage.

Salt stress can influence organic degradation by altering substrate properties and microbial activity [12] observed a decrease of 23% in pile porosity under salt stress (>0.6% NaCl), which suppressed ammonia-oxidizing bacteria, increasing ammonium nitrogen (NH_4_^+^-N) 2.4 times compared with the control. Moreover, the effects of salt stress on microbial communities often exhibit spatial heterogeneity within the compost piles [13]. Consequently, it is plausible to hypothesize that salinity not only affects the survival rates, oxidative stress responses, and mucus secretion of earthworms but also alters the composition of their gut microbiota, which, in turn, may impair the earthworm’s functional capacity for KW treatment. Furthermore, the disruptive effects of salt stress may reduce the efficiency of organic matter degradation during vermicomposting, thereby impacting the overall efficacy of the process. However, the precise mechanisms by which salinity influences microbial–earthworm interactions and the subsequent effects on the quality and stability of the final KW vermicomposting product remain poorly understood and warrant further investigation.

Therefore, the objectives of this study were to investigate the effects of salt stress on earthworm physiology and the quality of vermicompost products of KW and to reveal the underlying mechanisms. To achieve these objectives, the impacts of 35-day sustained salt stress (1.5% NaCl) on growth, survival, mucus secretion, stress enzyme activity, and gut microbiota were evaluated to understand the physiological responses of earthworms. Concurrently, the organic matter decomposition was measured with three-dimensional fluorescence spectroscopy to evaluate the vermicompost quality. In addition, the shifts in microbial community across upper and lower vermicompost layers were analyzed to elucidate the regulatory mechanisms of salt stress.

## 2. Materials and Methods

### 2.1. Experimental Materials

KW was prepared from fresh vegetables and fruits obtained from Hualian Supermarket, Anning District, Lanzhou City. Based on the local dietary habits, the KW consisted of watermelon rind, potato peels, lettuce, and cucumber in a wet weight ratio of 3:2:2:1. All fresh edibles were cut into ~5 mm pieces, soaked in deionized water for 12 h, thoroughly rinsed to remove salts, and homogenized. Refined iodized salt (99.1% NaCl; China Salt Group, Beijing, China) was added to the KW to simulate salinity stress. To ensure a single-variable condition, no additional interfering substances (e.g., fats) were added. Adult *Eisenia fetida* with clitella (~0.5 g fresh weight) were selected and acclimated for one week in a mixture of earthworm castings and KW. Earthworms, which exhibited active behavior and weight gain, were used for vermicomposting. The vermicomposting system consisted of a perforated plastic composting bucket with a fine mesh bottom. The bucket was integrated into a leachate collection system. From top to bottom, the system comprised (i) an expanded clay layer, (ii) a KW layer, (iii) a worm habitation layer, and (iv) a leachate collection layer. Table 1 shows the physicochemical properties of the initial KW and bedding substrate.

### 2.2. Experimental Setup

Two treatment groups were established: (i) control with no added salt (CK) and (ii) salt treatment group (ST) with 1.5% NaCl (*w*/*w*, based on wet weight). The salinity levels were established based on the range of salt content of KW in China, previous studies by Xia et al. [6], and the maximum tolerance of earthworms to NaCl (2%) determined in preliminary experiments. Each treatment had three replicates. Each reactor contained 3 kg of bedding substrate, with 500 g of KW added on days 0, 15, and 25. Expanded clay granules were applied to the surface of the bedding substrate to deter insect interference. In the ST group, KW was pre-mixed with 1.5% NaCl to simulate cumulative long-term salt stress. After adding KW, each reactor was inoculated with 300 *Eisenia fetida* individuals (~0.5 g each) and then covered with black cloth to maintain dark conditions. The temperature of the reactor was kept at 22 ± 1 °C. No additional feedstock was added during the experiment. Vermicomposting was terminated after 35 days, when no residual waste was visible on the surface of the bedding substrate. Samples were collected from the surface and bottom bedding layers. Surviving earthworms were counted and weighed. Subsequently, 30 earthworms were dissected for the extraction of intestinal content. All substrate samples (*n* = 3 per layer) were sealed in sterile plastic bags. One portion of the sample was used immediately for moisture and organic matter analysis. Another portion was air-dried, ground, sieved (65 mesh), and stored at 4 °C for analysis of salt content and physicochemical properties. The third portion was stored fresh and used for DNA processing. Earthworm intestinal samples collected after dissection were stored at −80 °C and used for DNA extraction.

### 2.3. Analytical Methods

#### 2.3.1. Analysis of the Physicochemical Properties in Vermicomposting Production

Moisture content and organic matter in the compost samples were determined gravimetrically. The samples were oven-dried at 105 °C to constant weight for moisture determination. To determine the organic matter content, dried samples were burned at 650 °C to constant weight. For pH and electrical conductivity (EC) measurements, air-dried samples were mixed with deionized water (1:50 *w*/*v*) and stirred at 300 rpm for 30 min. The pH and EC values of this suspension were measured using a pH meter (PHS-3C, Leici, Shanghai, China) and a conductivity meter (DDS-307, Leici, Shanghai, China), respectively. The mixture was centrifuged at 4000 rpm for 20 min (TGL 16M, Changsha, China), and the supernatant was filtered through a 0.45 µm membrane. Nitrate (NO_3_^−^–N) content was measured using the ultraviolet spectrophotometer (UV-3100, MAPADA, Shanghai, China). Total nitrogen (TN) content was measured using the alkaline potassium persulfate digestion and ultraviolet spectrophotometric method. Total phosphorus (TP) content was determined using the ammonium molybdate spectrophotometric method. Ammonia nitrogen (NH_4_^+^-N) content was determined using the spectrophotometric method with Nessler’s reagent, and the measurement was performed using a multiparameter water quality analyzer (CNPN-7SII, Hangzhou, China). Filtrates of 10-fold diluted samples were analyzed using a TOC analyzer (Multi N/C 2100, Analytik, Jena, Germany) to determine the dissolved organic carbon (DOC).

#### 2.3.2. Three-Dimensional Fluorescence Spectroscopy and Fourier Transform Infrared Spectroscopy

After adjusting the DOC concentrations of all samples to a standardized level, the 3D fluorescence emission matrix (3D-EEM) of the mixed solution was obtained using a fluorescence spectrophotometer (F-7100, Shimadzu, Kyoto, Japan), with excitation wavelength range of 220–450 nm, emission wavelength range of 220,500 nm, slit width of 5 nm, scan speed of 12,000 nm/min, and PMT voltage of 700 V. Samples were oven-dried at 80 °C for 2 h, mixed (1.0 ± 0.2 mg) with 100 mg spectroscopic-grade potassium bromide (KBr), and pressed into pellets. Fourier transform infrared spectroscopy (FTIR) (Bruker VERTEX 70, Bruker, Bremen, Germany) was used to record the FTIR spectra at a resolution of 4 cm^−1^ over the range of 4000–400 cm^−1^.

#### 2.3.3. Earthworm Intestinal Content Extraction and Mucus Extraction

Thirty earthworms were collected from each replicate reactor. The earthworms were rinsed in sterile 0.9% NaCl, cooled on ice for 10 min, euthanized in 70% ethanol, and dissected aseptically. Intestinal contents were pooled per replicate (*n* = 3) and stored at −80 °C. Following overnight fasting, earthworms were rinsed, dried, and subjected to three electrical stimulations (5 V, 60 s) at regular intervals of 60 s. Volume of mucus was measured, and then mucus was centrifuged (3000 rpm, 30 min) and filtered (0.22 µm) for analysis.

#### 2.3.4. Determination of Catalase and Superoxide Dismutase Activities

Catalase (CAT) activity was measured using the spectrophotometric method, whereas sample preparation followed Yang et al. [14] with minor modifications. Earthworm tissues (0.5 g) were homogenized in 2–3 mL of ice-cold phosphate buffer (0.2 mol/L, pH 7.0) with quartz sand, and the homogenate was brought to a final volume of 25 mL. After standing on ice for 10 min, the homogenate was centrifuged at 4000 rpm for 15 min at 4 °C to obtain the enzyme extract. For the blank control, 1 mL of this extract was heat-inactivated by boiling for 5–10 min and rapidly cooled on ice to correct for non-enzymatic H_2_O_2_ degradation. For the assay, 1 mL of untreated extract was mixed with freshly prepared H_2_O_2_, 5 mL phosphate buffer (pH 7.8), and 5 mL distilled water, and the decrease in absorbance at 240 nm was recorded for 1 min using a UV-visible spectrophotometer. CAT activity was calculated from the linear rate of H_2_O_2_ degradation (ΔA_240_/min) and expressed as U/mg.

Superoxide dismutase (SOD) activity was determined using the method described by [14] with slight modifications. A 5 mL aliquot of the tissue homogenate was centrifuged at 10,000 rpm for 10 min. The supernatant was collected as the crude SOD extract and stored at 4 °C. To measure the SOD activity, 3 mL of phosphate buffer (0.05 mol/L, pH 7.8) was added to 0.6 mL of each of the following solutions: Met solution (130 mmol/L), NBT solution (750 μmol/L), EDTA-Na_2_ solution (100 μmol/L), and riboflavin (20 μmol/L). Then, 0.1 mL of the crude SOD extract and 0.5 mL of distilled water were added to this mixture. In the control group, phosphate buffer was used instead of the crude enzyme solution. The final volume of this mixture was adjusted to 6 mL, and the absorbance was measured at 560 nm.

#### 2.3.5. DNA Extraction and High-Throughput Sequencing

DNA was extracted from 1 g of fresh vermicomposting production sample using the TIANamp Soil DNA Kit (Tiangen, Beijing, China). DNA concentration was measured with a Qubit fluorometer (Thermo Fisher Scientific, Waltham, MA, USA) to ensure ≥24 ng/µL concentration for downstream applications. The V3-V4 region of the 16S rRNA gene and the V4 region of the 18S rRNA gene were amplified using primers 341F/806R and 528F/706R, respectively, PCR reactions contained 15 µL Phusion High-Fidelity PCR Master Mix, 0.2 µM of each primer, and approximately 10 ng of DNA template, using the following cycling conditions: initial denaturation at 98 °C for 1 min; 30 cycles of 98 °C for 10 s, 50 °C for 30 s, and 72 °C for 30 s; and a final extension at 72 °C for 5 min. Amplicons were purified with AMPure beads and used to construct PCR-free sequencing libraries with the TruSeq^®^ DNA PCR-Free Library Preparation Kit (Illumina, San Diego, CA, USA). Library preparation followed the manufacturer’s PCR-free workflow, including end repair, adapter ligation, and library purification. The resulting libraries passed Qubit and qPCR quality checks and were sequenced on an Illumina NovaSeq 6000 platform (Novogene, Beijing, China). Raw reads were demultiplexed, trimmed, and merged with FLASH (v1.2.11); residual primers were removed with Cutadapt; and quality filtering was performed with fastp (v0.23.1). Chimeric sequences were identified using the SILVA database (release 138.1) and removed accordingly. Denoising and amplicon sequence variants (ASV) inference were conducted using DADA2 in QIIME2, and taxonomic assignment of 16S and 18S rRNA gene sequences was performed against SILVA138.1. The sequencing data are available in the NCBI SRA database under BioProject accession PRJNA1380645.

#### 2.3.6. Statistical Analysis and Data Visualization

Statistical analyses were performed using IBM SPSS Statistics 26, with a significance threshold of *p* < 0.05. The differences between samples were analyzed using *t*-tests and one-way ANOVA. A two-way ANOVA was performed to evaluate the main effects of salt treatment and layer, as well as their interaction. Data were visualized using Origin 2024, GraphPad Prism 9.5 and R version 4.4.3 (R Core Team, 2025). To analyze the 3D-EEM data, parallel factor analysis (PARAFAC) and fluorescence regional integration (FRI) were conducted using the MATLAB R2023b platform. For cluster heatmap generation (at the genus level) and principal component analysis (PCA) of the samples’ physicochemical properties, pheatmap, FactoMineR, and factoextra packages in R were employed. A neutral community model (NCM) was constructed by using the humic, minpack.lm, stats 4, grid and ggplot 2 packages in R. Partial least squares structural equation modeling (PLS-SEM) was performed using SmartPLS 4.

## 3. Results and Discussion

### 3.1. Effects of Salt Stress on the Physiological Characteristics of Earthworms

#### 3.1.1. Impact on Earthworm Biomass

In vermicomposting systems, the physiological condition of earthworms is a key determinant of vermicomposting efficiency and compost quality. As shown in Table 2, the number of earthworms in the CK group increased from 300 to 305 during the vermicomposting, while the average body weight increased from 0.549 g to 0.555 g, showing a growth rate of 2.8%. These results demonstrate that, under optimal conditions, earthworms maintain physiological homeostasis through balanced feeding and metabolism. In contrast, the ST group exhibited a sharp decline in earthworm number from 300 to 227, showing a mortality rate of 24.33% (*p* < 0.05). The average body weight of earthworm decreased to 0.324 g in the ST group, representing a significant reduction of 43.6% (*p* < 0.01). These results indicate that elevated salt concentrations elicit severe negative effects on the physiological condition of earthworms, significantly impairing their population stability and activity. Such stress-induced declines in earthworm biomass are closely linked to reduced treatment efficiency of the vermicomposting system [15].

**Figure 1:**
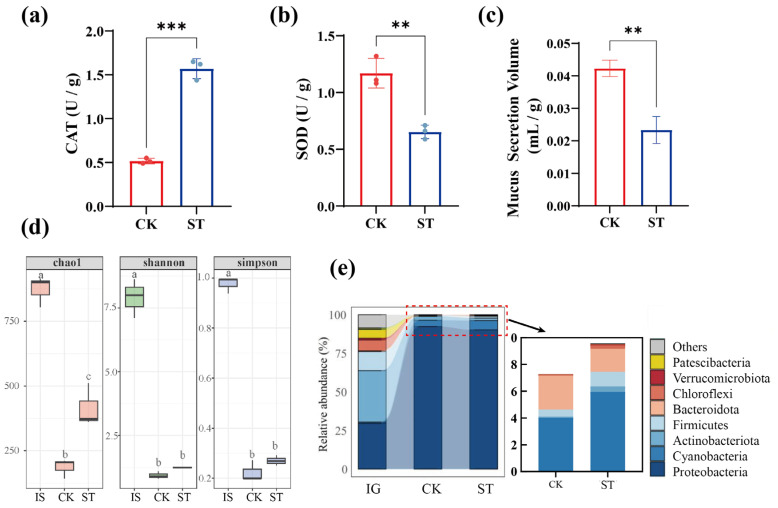
Effects of salt stress on earthworm biomass, antioxidant enzyme activity, and mucus production after 35 days of vermicomposting: (**a**–**c**) CAT activity, SOD activity, and mucus secretion in earthworms; (**d**) Alpha diversity indices of earthworm intestinal microbiota; (**e**) Phylum-level distribution of intestinal microbiota.* Indicates a significant difference between treatments, as determined by the *t*-test. * *p* < 0.05; ** *p* < 0.01; *** *p* < 0.001. Different letters (a, b, c) between treatments indicate significant differences between groups, as determined by the Tukey’s HSD test (*p* < 0.05).

As shown in Figure 1a, CAT activity increased significantly in the ST group after 35 days (*p* < 0.01), indicating that earthworms enhanced their hydrogen-peroxide scavenging capacity to maintain cellular redox balance and mitigate oxidative damage under high-salinity stress. In contrast, Figure 1b demonstrates a marked decline in SOD activity in the ST group (*p* < 0.01). Such a reduction typically reflects disruptions in reactive oxygen species (ROS) homeostasis, oxidative damage to the enzyme’s metal catalytic center, and protein conformational alterations induced by ionic or osmotic stress, ultimately impairing its catalytic function [16]. Notably, the asynchronous changes in CAT and SOD are consistent with the findings of Yang et al. [14]. This divergent response highlights the selective effects of ROS on the antioxidant system under salt stress: excessive superoxide renders SOD more susceptible to oxidative inactivation, whereas CAT is compensatorily upregulated due to its central role in H_2_O_2_ detoxification. Moreover, salt-induced ROS accumulation further disrupts antioxidant enzyme homeostasis, causing the more heavily loaded SOD to be preferentially impaired, thereby amplifying its divergence from CAT.

Mucus secretion typically increases under acute stress [17]. However, in this study, the earthworms in the ST group secreted significantly less mucus than those in CK (*p* < 0.01, Figure 1c). There may be two explanations for this reduction in mucus secretion. First, due to biomass loss, earthworm activity was reduced, thereby limiting the mucus production capacity. Second, epidermal tissue damage was caused by salt-induced ROS accumulation [14], which led to oxidation of epidermal and intestinal tissues [18,19], thereby impairing earthworm activity and secretory functions.

#### 3.1.2. Impact on Intestinal Microbiota

As shown in Figure 1d, the intestinal microbiota of the original earthworm exhibited significantly higher Chao1, Shannon, and Simpson indices than those in both CK and ST groups after 35 days (*p* < 0.01). This suggests that the earthworms progressively select symbiotic microbial communities with specialized ecological functions during vermicomposting, leading to a stable, niche-adapted microbiome. The Chao1 index in the ST group was 122.6% higher than that in the CK (*p* < 0.05), indicating that salt stress exerted a selective pressure favoring the growth of salt-tolerant bacterial taxa. This may have facilitated competitive colonization by opportunistic bacterial species, thereby increasing species richness in intestinal microbiota. Such shifts in microbial diversity likely resulted from altered intestinal osmotic pressure and metabolite composition, which created new ecological niches for previously less-abundant salt-tolerant bacteria [20]. However, no significant differences were observed in the Shannon or Simpson indices (*p* > 0.05) between the treatment groups, indicating that salt stress affected species richness rather than evenness. These results suggest that the expansion of salt-tolerant taxa was accompanied by a decline in salt-sensitive species. Figure 1e shows that the original intestinal microbiota was dominated by *Actinobacteria* (33.26%), *Proteobacteria* (29.69%), and *Firmicutes* (12.34%). The high abundance of *Actinobacteria* is likely linked to the lignocellulose-degrading capacity of earthworms [21]. After 35 days, both CK and ST groups exhibited dramatic shifts, with *Proteobacteria* abundance exceeding 90% and *Actinobacteria* abundance declining significantly (*p* < 0.01). This suggests that *Proteobacteria* occupy the core functional niche during the maturation phase of vermicomposting, as observed by [22]. Members of *Proteobacteria* contribute to organic matter mineralization and nitrogen cycling via extracellular hydrolytic enzymes [23], and they often show enrichment under environmental stress or shifts in substrate composition. Wang et al. [24] reported a positive correlation between *Proteobacteria* abundance and lignin degradation efficiency in a cow manure-based vermicomposting system, supporting their role in the transformation of complex organic matter. Therefore, the preferential enrichment observed here may represent an adaptive response of intestinal microbiota to metabolic pressures caused by vermicomposting substrates.

No significant differences in phylum-level composition of intestinal microbiota were observed between CK and ST groups (*p* > 0.05). This is common in sub-lethal stress conditions and may reflect the ability of earthworms to regulate coelomic osmotic pressure, stabilizing gut conditions and enabling tolerance to moderate salt exposure. The salt stress in the ST group may have been insufficient to drive major phylum-level shifts in the composition of intestinal microbiota over the experimental period, especially given their inherent resilience and stability under moderate stress.

### 3.2. Effects of Salt Stress on Substrate Characteristics

#### 3.2.1. Impact on Physicochemical Properties of the Upper and Lower Layers

**Figure 2:**
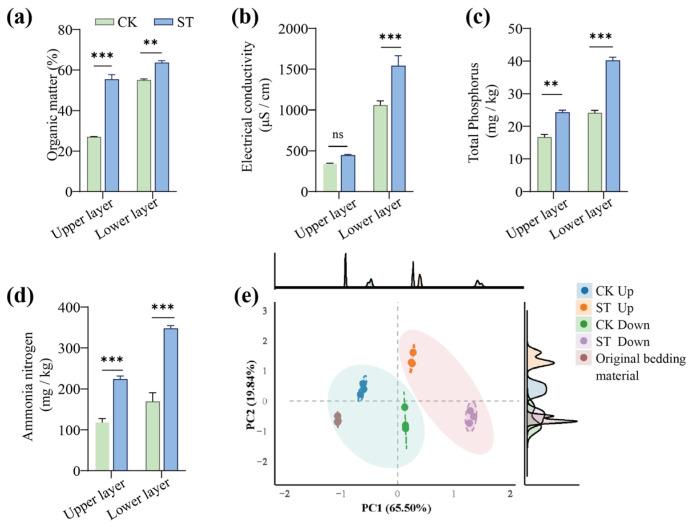
Effects of salt stress on substrate properties: (**a**–**d**) Physicochemical characteristics of the upper and lower substrate layers in CK and ST groups; (**e**) PCA of the physicochemical properties of the original bedding substrate and the samples collected from the upper and lower layers of CK and ST after vermicomposting. * Indicates a significant difference between treatments, as determined by the *t*-test. * *p* < 0.05; ** *p* < 0.01; *** *p* < 0.001; ns indicates no significant difference (*p* > 0.05).

After 35 days of vermicomposting, the cumulative salt concentrations in the upper and lower substrate layers reached 3.2% and 1.8%, respectively, in the ST group. As shown in Figure 2a, the organic matter content in the upper layer of the CK group was 94.64% lower compared to the ST (*p* < 0.001). This suggests that elevated salinity inhibits earthworm feeding activity, leading to the accumulation of undecomposed organic matter in the surface layer. In the lower layer, vertical migration of earthworms mechanically disturbs the substrate, incorporating undecomposed organic matter from the surface layer. However, under salt stress, the imbalanced osmotic pressure reduces the substrate decomposition efficiency of the vermicomposting system, promoting the accumulation of unprocessed material in the lower layer. Consequently, the ST group exhibited significantly higher organic matter content (14.86%) in the lower substrate layer compared with the CK (*p* < 0.01). Figure 2b shows that the EC in the lower substrate layer was 45.53% higher in the ST group compared with the CK (*p* < 0.01), whereas no significant differences were observed in the upper layer (*p* > 0.05). In the ST group, poor decomposition of KW led to downward migration of salt ions via leachate, resulting in the accumulation of salt ions at the bottom of the reactor.

Similarly, Figure 2c,d shows that the TP contents in the upper and lower layers of the ST group were 47.06% and 66.58% higher, respectively, compared with the CK (*p* < 0.001). Similarly, NH4^+^-N contents were 93.97% and 115.46% higher in the upper and lower layers of ST, respectively, compared with the CK (*p* < 0.001). These patterns indicate that salt stress can disrupt the synergistic degradation functions of earthworms and microorganisms, causing accumulation of organic matter and salts in the surface layer while driving migration of ions to the lower layer. The resulting osmotic pressure gradient suppresses the activities of microbes and earthworms in the deeper layers of the vermicomposting system, reducing overall organic matter degradation efficiency and ultimately affecting the quality of vermicompost. Figure 2e presents the PCA results, showing a clear separation (*p* < 0.01) between the CK and ST groups. Original substrate samples and CK upper-layer samples clustered along the negative PC1 axis (PC1: 65.50%), whereas ST upper-layer samples shifted toward the positive PC1 axis. The lower-layer samples from the ST group exhibited the greatest divergence from other samples. These findings indicate that salinity induces a global restructuring of substrate microenvironment, altering the metabolic processes driving organic matter degradation. The spatial distribution patterns observed in this study provided multidimensional evidence of metabolic imbalance under salt stress, offering valuable insights for optimizing the vermicomposting process by managing the key physicochemical properties of production.

#### 3.2.2. Effects of Salt Stress on the Humification of Organic Matter

**Figure 3:**
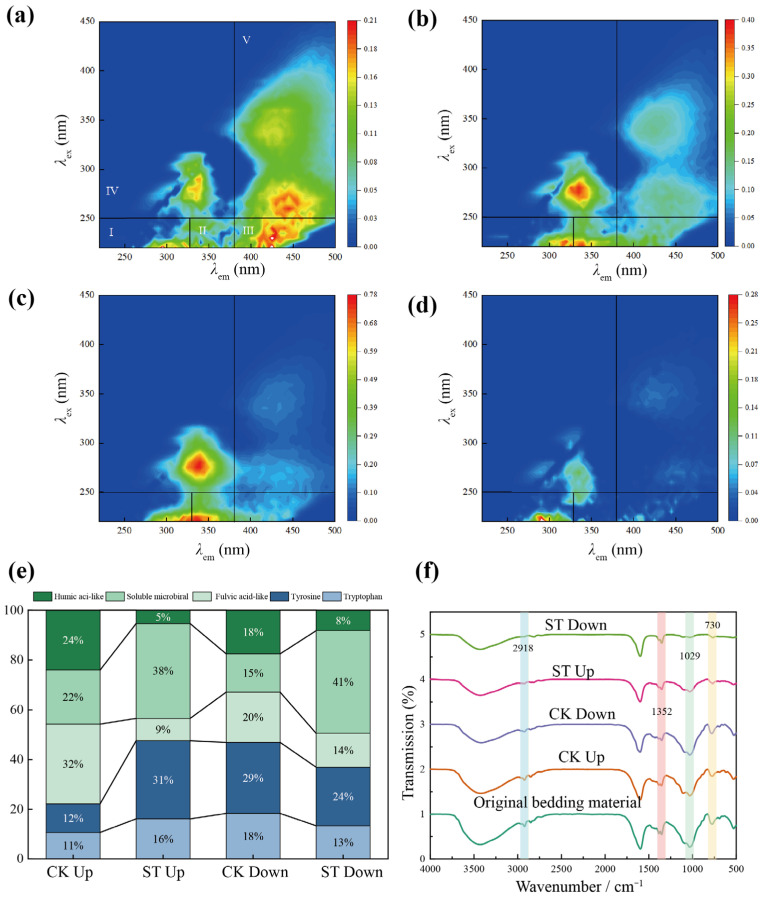
Effects of salt stress on substrate humification: (**a**–**d**) Three-dimensional fluorescence spectra of DOM in upper and lower substrate layers; (**e**) Fluorescence region integration of the spectra of both layers; (**f**) FTIR spectra of the upper and lower layers.

After 35 days of vermicomposting, 3D-EEM revealed the distinct differences in the humification patterns observed in the CK and ST groups. As shown in Figure 3a, the fluorescence peaks in the 3D-EEM of the CK upper-layer sample were primarily concentrated in regions III and V, with a smaller peak in region IV. This distribution indicates the formation of humic substances and continued microbial metabolic activity in the earthworm casts, which contributed to organic matter degradation. In contrast, the CK lower-layer sample exhibited peaks mainly in regions I and II, with minor peaks in regions III and V (Figure 3b), suggesting higher proportions of incompletely decomposed proteins and limited synthesis of molecular-weight humic substances.

As shown in Figure 3c, the fluorescence peaks in the upper layer of the ST group were concentrated in regions I, II, and IV, indicating substantial retention of partially degraded proteins due to reduced microbial activity. The strong peak in region IV suggests the persistence of salt-tolerant microorganisms, which potentially produce extracellular polymeric substances or metabolites to maintain the osmotic balance. Figure 3d shows that the lower layer of ST exhibited dominant peaks in region IV, reflecting vertical bioturbation by earthworms that caused downward transport of undecomposed proteins and secreted compounds. These findings indicate that cumulative salt stress inhibits humification in the upper layer, promotes accumulation of undegraded proteins, and shifts microbial metabolism toward osmotic regulation rather than organic matter decomposition.

As depicted in Figure 3e, humic acid content in the upper layer of ST (5.34%) was significantly lower than that in CK (24.02%) (*p* < 0.01). Similarly, fulvic acid content declined by 71.1% in the ST group, suggesting suppression of phenol–quinone oxidative polymerization [25]. In contrast, contents of undegraded aromatic proteins (16.12%) and microbial metabolites (31.44%) were significantly higher than those in the CK (*p* < 0.05). This indicates that high salinity levels redirect microbial metabolism toward the production of extracellular polymeric substances for osmotic balance [26]. In the lower layer of ST, the concentration of soluble microbial products was 2.7 times higher than that in the CK, indicating adaptive metabolic reorganization in anaerobic microbes. However, humic acid content declined by 53.2% in the ST group, which confirmed that the inhibitory effects of salt on humification extended throughout the substrate. Stratification analysis revealed that the upper layer in the ST was dominated by undegraded proteins (PI + PII = 47.56%) and soluble microbial products (PIV = 38.11%). On the other hand, the lower layer contained an even higher proportion of soluble microbial products and a slightly higher humic acid content, likely due to ion migration, dilution effects, and microbial community shifts.

Humification index (HIX) values further supported these findings (Figure S1). The HIX in the upper layer of ST was significantly lower than that in the CK (*p* < 0.01), indicating a shift from stable humic forms toward non-humified organic matter. These results aligned with the reductions in humic and fulvic acid contents revealed using EEM, confirming that salt stress disrupted phenolic condensation and quinone polymerization [27]. The β:α index also declined under salt stress, indicating reduced contribution of microbial autotrophic production and increased proportion of terrestrial or undecomposed organic matter. The increased soluble microbial products and lower β:α index suggest that salt-tolerant microbes produce more metabolites with simple chemical structures, hindering the formation of complex humification precursors. Although the HIX in the lower layer of ST was higher than that in the upper layer, it remained significantly below that in the CK (*p* < 0.01), suggesting partial microbial adaptation but overall reduced stability of humus.

As illustrated in Figure 3f, FTIR spectra further revealed chemical alterations in humic structures. The original substrate showed strong peaks at 730 cm^−1^ and 1029 cm^−1^. After vermicomposting, peak intensity at 730 cm^−1^ decreased in both layers of CK, indicating the degradation of aromatic residues by microbes and earthworms [28]. Salt stress further altered the degradation pathway. Although the peak at 730 cm^−1^ also declined in the ST group, the accompanying decrease in β:α index indicates the inhibition of lignin-degrading microbes, which limited the conversion of aromatic compounds into humic acid precursors.

The peak at 1029 cm^−1^ decreased further in the FTIR spectrum of the ST group, suggesting the degradation of structural polysaccharides and possible modification of extracellular polysaccharides of microorganisms [29]. This pattern aligned with the higher level of soluble microbial products and lower β:α values, implying secretion of low-complexity polysaccharides by microorganisms for osmotic regulation. The peaks at 1430 cm^−1^ and 2918 cm^−1^, corresponding to the aliphatic compounds, also reflected altered humification dynamics. In the CK group, the intensities of these peaks decreased markedly due to the conversion of complex organic compounds into humic acid. In the ST group, the peak intensity at 1430 cm^−1^ increased, while the peak at 2918 cm^−1^ continued to decline, suggesting inhibition of oxidase activity and impaired condensation of short-chain aliphatic compounds into humic structures [30]. Such inhibition may result from ionic interference with hydrophobic interactions in humic acid microdomains [31,32].

Spatial analysis showed that the intensity of the 1430 cm^−1^ peak in the lower layer of ST was higher than that in the upper layer and slightly lower than that at 2918 cm^−1^, indicating that lower-layer microbes partially delayed salt-induced inhibition of aliphatic compound conversion. However, the humification capacity of microbes remained substantially impaired. In summary, cumulative salt stress inhibited the humification of earthworm casts via three primary mechanisms: (1) suppression of aromatic compound conversion; (2) disruption of aliphatic condensation; and (3) simplification of polysaccharide structures. These alterations reduced the humus stability and shifted organic matter conversion toward more labile but less recalcitrant forms.

#### 3.2.3. Effects of Salt Stress on the Microbial Communities in the Substrate

**Figure 4:**
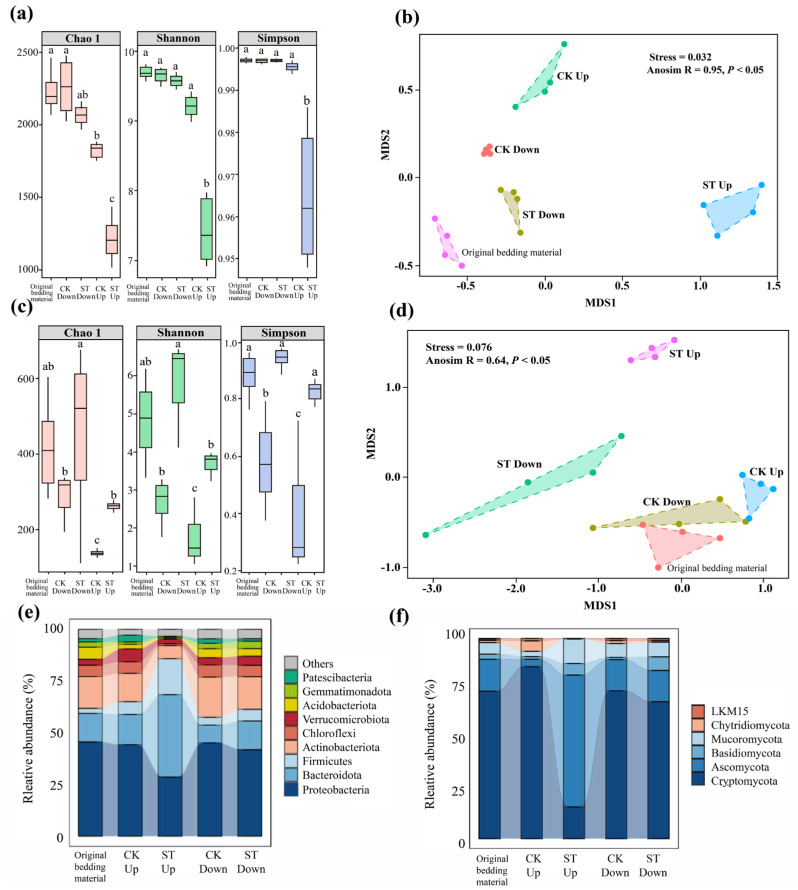
Alpha and beta diversity of bacteria (**a**,**b**) and fungi (**c**,**d**) in the upper and lower substrate layers, and their abundance at the phylum level for bacteria (**e**) and fungi (**f**) in both layers. Different letters (a, b, c) between treatments indicate significant differences between groups, as determined by the Tukey’s HSD test (*p* < 0.05).

As shown in Figure 4a, the Shannon, Chao1, and Simpson indices in the lower layer of the ST were significantly lower (*p* < 0.05) than those in the CK, indicating reduced microbial diversity and evenness under high-salt conditions. β-diversity analysis revealed distinct clustering of upper-layer samples and clear separation of these samples from lower-layer samples in the ST group (Figure 4b). This indicates that high salinity exerted differential selective pressures across vertical strata, substantially reshaping the composition of microbial communities in the substrate.

As depicted in Figure 4e, after 35 days of vermicomposting, the bacterial community in the upper layer of CK was dominated by *Proteobacteria* (44.30%), followed by Bacteroidetes (14.60%), *Actinobacteria* (13.70%), and *Firmicutes* (6.22%). This composition aligned with the typical vermicomposting systems, where the members of *Proteobacteria* play a primary role in mineralizing complex organic matter via diverse metabolic pathways [33,34]. Furthermore, *Actinobacteria* and *Firmicutes* contribute to lignocellulose and chitin degradation [17,35], while Bacteroidetes efficiently degrade the polysaccharides [36]. In the ST group, abundance levels of Proteobacteria, Actinobacteria, and Chloroflexi declined significantly (*p* < 0.05), whereas Bacteroidetes and *Firmicutes* abundance increased significantly (*p* < 0.01), indicating persistent selective pressure and major shifts in microbial community structure.

At the genus level (Figure S2), the upper layer of CK was dominated by *Flavobacterium* (7.08%), *Acinetobacter* (2.44%), and *Algoriphagus* (3.35%). *Flavobacterium* facilitates rapid degradation of soluble organic matter by exhibiting high activity of extracellular hydrolase [37]. In the ST group, *Trichococcus* (3.05%), *Pseudorhodobacter* (2.87%), and *Flavobacterium* (1.96%) were the dominant genera. *Trichococcus* can tolerate high salinity by accumulating betaine-compatible solutes and metabolizing small sugars [34], while *Pseudorhodobacter* may adapt to salt stress via light-driven proton pumps that regulate ion gradients [38]. The enrichment of *Bacteroides* (2.82%) and *Cytophaga* (1.54%), which can degrade pectin and cellulose [39], may partially offset the reduced carbon conversion efficiency of the vermicomposting system resulting from the decline in *Proteobacteria* abundance. Notably, salt treatment introduced anaerobic genera, such as *Acetoanaerobium* (1.87%) and *Marinobacterium* (2.50%), suggesting salt-induced anoxia, with these taxa contributing to acetate fermentation and sulfur reduction [40]. Increased abundance levels of *Lactococcus* (1.92%) and *Proteiniclasticum* (1.77%) indicate enhanced lactic acid fermentation and protein hydrolysis. Conversely, the decrease in Chloroflexi abundance likely reduced lignin conversion efficiency, consistent with the inhibition of humic acid-like substances (Zone V) in 3D-EEM spectra and the persistence of aromatic residues (730 cm^−1^) in FTIR spectra. Although salt-tolerant microbial taxa maintained organic matter decomposition through metabolic adjustments, the reduced complexity of their products may lower the fertility of earthworm casts.

Fungal α-diversity patterns showed significantly higher Shannon and Chao1 indices in the lower layer of ST, as compared to CK (*p* < 0.05, Figure 4c). The Simpson index also showed a similar trend, indicating an increase in fungal richness under salt stress. This may relate to the vertical migration of salt ions. Non-metric multidimensional scaling (NMDS, Figure 4d) analysis revealed that the upper-layer and lower-layer samples of CK were clustered closely with the original substrate. The ST group displayed marked dissimilarity, with the lower-layer samples being distant from the CK cluster and the upper-layer samples being entirely separated, indicating strong vertical stratification of salt effects.

As shown in Figure 4f, the fungal community in the ST group was dominated by *Cryptomycota* (73.82%), followed by *Ascomycota* (15.91%), *Mucoromycota* (5.67%), and *Basidiomycota* (2.44%). The fungal species of the Cryptomycota phylum, likely benefiting from their saprophytic lifestyle [41], participate in the early-stage decomposition of substrate. Mortierella, a key genus in Mucoromycota phylum, can degrade lipids and insect residues by secreting lipase and chitinase [42]. After vermicomposting, Cryptomycota abundance in the upper layer of CK increased to 86.10%, while Ascomycota abundance significantly reduced (*p* < 0.05). In contrast, the microbial community in the upper layer of the ST group was dominated by *Ascomycota* (66.01%), followed by *Cryptomycota* (15.78%), *Mucoromycota* (12.06%), and *Basidiomycota* (5.78%) (Figure S3). This shift in microbial community was driven by Geotrichum and Galactomyces. The members of Geotrichum likely tolerate salt stress through glycerol transporter expression [43], while Galactomyces can degrade lactose-containing substrates via halophilic β-galactosidases [44]. Mucor abundance (9.82%) showed a 3.2-fold increase in the ST group (*p* < 0.01), potentially due to salt-tolerant spores and preference for short-chain fatty acids [45]. The reduced abundance of Cryptomycota and Mortierella hindered the degradation of chitin and lignin–cellulose complexes, consistent with suppressed humic acid formation and elevated intensity of peaks at 1430 cm^−1^ in FTIR spectra, suggesting disrupted humification under salt stress via functional community shifts.

There were no significant differences between treatment groups in terms of fungal community in the lower substrate layer (*p* > 0.05). Furthermore, the compositions of these fungal communities resembled those in the original substrate. Stable abundance levels of *Mortierella* (15.23%) and *Chaetomium* (8.91%) suggest that the low oxygen in the lower layer supported the activity of lignin-modifying enzymes, with this effect potentially influenced by salt ion leaching and osmotic gradients. Overall, the effects of salinity were spatially constrained. The upper layer in ST experienced strong selective pressures and major functional shifts, while the lower layer remained relatively stable. The enrichment of Ascomycota enhanced the resilience of the vermicomposting system under salt stress. However, the loss of key saprotrophs, such as Cryptomycota, reduced the degradation of recalcitrant organic matter. Future metagenomic analyses are needed to elucidate the functional genes of halotolerant Ascomycota and assess their effects on microbial interactions and vermicompost quality.

### 3.3. Mechanisms Underlying the Effects of Salt Stress on the the Vermicomposting System

**Figure 5:**
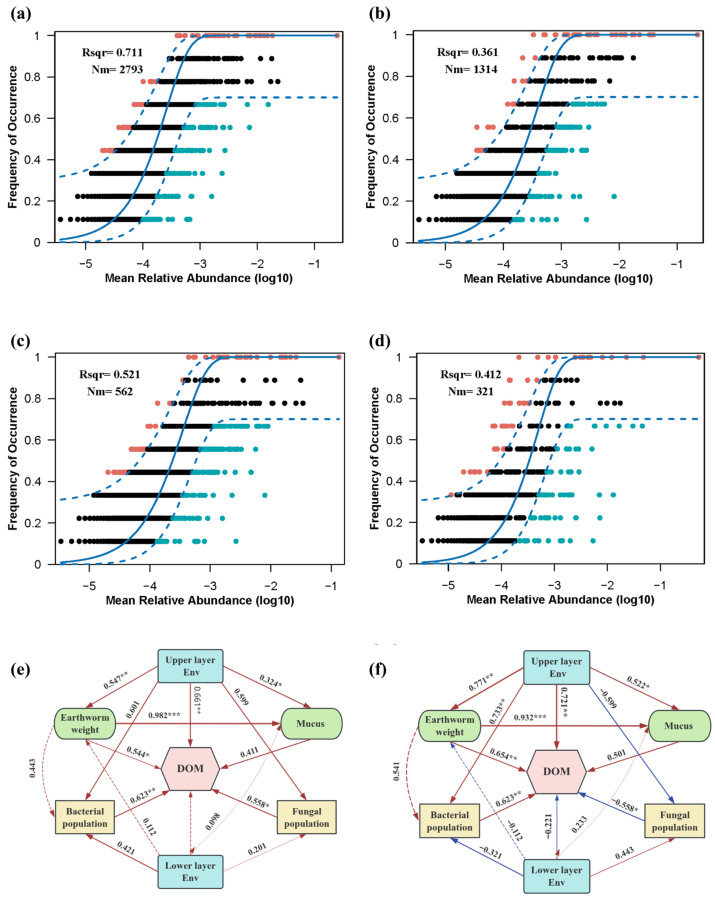
Mechanisms of salt stress affecting the vermicomposting systems: (**a**,**b**) Bacterial NCMs for CK and ST groups, respectively; (**c**,**d**) Fungal NCMs for CK and ST groups, respectively; and (**e**,**f**) PLS-SEM illustrating the influence mechanisms of salt stress. The arrow thickness indicates the strength of the relationship between variables, with thicker arrows representing stronger effects. Red arrows denote positive relationships, while blue arrows represent negative relationships. The numbers next to the arrows represent the path coefficients, reflecting the magnitude of the effects. Solid lines indicate statistically significant effects (* *p* < 0.05; ** *p* < 0.01; *** *p* < 0.001), while dashed lines represent non-significant relationships (*p* > 0.05).

As shown in Figure 5a,b, NCM analysis revealed distinct shifts in microbial community assembly processes under salt stress. CK group exhibited a strong neutral fit (R^2^ = 0.711, m = 0.17), indicating that stochastic processes, primarily random dispersal and ecological drift, were dominant drivers of bacterial community assembly in this group. In contrast, the ST group showed a markedly reduced neutral fit (R^2^ = 0.361, m = 0.08), reflecting an increased role of deterministic selection in community assembly. This shift likely resulted from the following complementary mechanisms. First, elevated salinity imposed physicochemical stress via Na^+^ and Cl^−^ ions, compromising the membrane integrity of salt-sensitive Gram-negative bacteria and conferring competitive advantages to halotolerant groups, such as *Pseudomonas* spp. [46]. Second, salt stress impairs the electron transfer processes of salt-sensitive microorganisms, including both extracellular and intracellular electron flow, thereby driving a shift in the microbial community toward a more energy-efficient direct interspecies electron transfer pathway. This shift preferentially enriches microbial populations with enhanced electron transfer capabilities and greater salt tolerance, thereby altering the microbial community structure [47]. Furthermore, it enhances the functional potential of specific microbial groups, facilitating the growth of microbes capable of overcoming both ionic stress and limitations in electron transfer. Finally, increased salt accumulation in the lower substrate layer led to earthworms exhibiting avoidance behavior, which in turn reduced their migration and bioturbation activities. The limited earthworm movement restricts stochastic dispersal, thereby facilitating local colonization by salt-tolerant bacteria [14]. Collectively, these findings indicate that salinity imposes a strong selective filter and restructures the functional potential of bacterial communities.

Fungal communities responded differently to salt stress (Figure 5c,d). While deterministic selection was evident, partial signatures of stochastic dispersal persisted, likely due to the intrinsic dispersal capacity of fungal hyphae, which can extend locally, even under reduced earthworm activity. Nevertheless, salt stress significantly altered the composition of fungal communities. The relative abundance of Ascomycota increased from 54.3% to 72.1%, whereas the abundance of Basidiomycota, which are key decomposers of humic substances, declined sharply. These patterns suggest that salt stress shifted fungal community composition toward taxa with greater halotolerance but potentially reduced the humification capacity.

As shown in Figure 5e, PLS-PM further demonstrated the significant direct effects of earthworm weight, upper-layer environmental factors, and microbial population on DOM transformation in the CK group (path coefficients = 0.544, 0.661, and 0.623, respectively; *p* < 0.01). Lower-layer environmental factors did not directly enhance humification but promoted microbial growth and earthworm activity via bioturbation. Figure 5f and Figure S4 show that, under cumulative salt stress, humification efficiency remained closely associated with earthworm weight (path coefficients = 0.654, *p* < 0.01), upper-layer environmental factors (path coefficients = 0.721, *p* < 0.01), and bacterial population (path coefficients = 0.623, *p* < 0.01). Additionally, earthworm weight exerted indirect effects on humification by stimulating epidermal mucus secretion (path coefficients = 0.932, *p* < 0.001), which itself was regulated by upper-layer environmental factors (path coefficients = 0.522, *p* < 0.05). The greater salt accumulation in the upper layer of the ST group likely caused direct earthworm epidermal irritation, triggering decreased mucus production.

Vertical stratification of environmental conditions of substrate significantly influenced both microbial population and earthworm weight in the ST treatment (*p* < 0.01), underscoring the role of spatial heterogeneity in shaping microbial community structure and driving biomass dynamics. Overall, salt stress altered the humification efficiency of the vermicomposting system through a three-dimensional regulatory network integrating biological adaptation (earthworm physiological responses), physicochemical gradient maintenance (ion balance and conductivity), and microbial functional compensation (enrichment of halotolerant taxa).

This multifactorial synergy highlights that the coupled biological, chemical, and microbial processes supported the vermicomposting under salinity stress. Overall, this study provided a theoretical framework for optimizing the treatment of highly saline KW.

## 4. Conclusions

High salt content in KW suppressed earthworm activity during vermicomposting, resulting in divergent CAT and SOD responses to oxidative stress, reduced epidermal mucus secretion, and adaptive restructuring of the intestinal microbiota with enrichment of halotolerant taxa. In the upper layer, inhibited humification favored Ascomycota while decreasing saprotrophic Basidiomycota. In the lower layer, microbial community assembly was shaped by salt accumulation and earthworm bioturbation. Bacterial communities shifted toward deterministic selection, whereas fungal communities retained partial stochasticity. Overall, under salt stress, the vermicomposting system reached a dynamic but functionally compromised equilibrium through earthworm physiological adaptation, physicochemical stratification, and microbial functional compensation. Although halotolerant bacteria maintained core decomposition processes, reduced metabolic product diversity impaired vermicomposting efficiency and consequently lowered the quality and fertility of the final vermicompost.

## Figures and Tables

**Table 1:** Physicochemical properties of initial bedding material and KW used in the study (mean ± SD, *n* = 3).

	Initial Bedding Materials	Kitchen Wastes
Organic matter (%)	29.47 ± 0.31	94.41 ± 0.51
Water content (%)	56.42 ± 0.63	92.32 ± 0.32
pH	7.35 ± 0.02	5.59 ± 0.28
Electrical conductivity (μS/cm)	230.5 ± 2.69	1911.1 ± 3.22
Nitrate (mg/kg)	235.65 ± 34.21	2303.16 ± 44.26
Total nitrogen (mg/kg)	767.85 ± 49.92	13,655.29 ± 301.35
Total phosphorus (mg/kg)	0.209 ± 0.046	76.93 ± 3.73
Ammonium (mg/kg)	82.83 ± 4.33	13,016.21 ± 441.33

**Table 2:** Biomass of earthworms before and after vermicomposting.

	0 d			35 d		
	Weight (g)	Number	Average Weight (g)	Weight (g)	Number	Average Weight (g)
CK	164.7	300	0.549	169.4	305	0.555
ST	172.5	300	0.575	73.5	227	0.324

## Data Availability

The original contributions presented in this study are included in the article/Supplementary Material. Further inquiries can be directed to the corresponding author.

## Supplementary Materials

The following supporting information can be downloaded at https://www.mdpi.com/article/10.3390/bioengineering13010038/s1, Figure S1: Fluorescence characteristic parameters of the upper and lower layers of the substrate.; Figure S2: The abundance of bacteria at the genus levels in the upper and lower layers of the substrate.; Figure S3: The abundance of fungi genus levels in the upper and lower layers of the substrate.; Figure S4: Partial least squares path modeling (PLS-PM) revealed the total effects under salt stress on the vermicomposting system.

## Abbreviations

The following abbreviations are used in this manuscript:

ST	Salt Treatment
KW	Kitchen waste
CK	Control group
