# Supplementary material for: Effects of Salt Stress on Earthworm Function and Compost Quality During Vermicomposting of Kitchen Wastes
Source: Bioengineering (Basel). 2025 Dec 29;13(1):38. doi: 10.3390/bioengineering13010038 (PMC12837463; doi:10.3390/bioengineering13010038)
Supplement: Supplementary file 1 [file bioengineering-13-00038-s001.zip › bioengineering-3966601-supplementary.pdf]

Supplemental materials for

Effects of Salt Stress on Earthworm Function and Compost Quality during Vermicomposting of Kitchen Wastes

Captions of table and figures

Fig. S1. Fluorescence characteristic parameters of the upper and lower layers of the substrate.

Fig. S2. The abundance of bacteria at the genus levels in the upper and lower layers of the substrate.

Fig. S3. The abundance of fungi genus levels in the upper and lower layers of the substrate.

Fig. S4. Partial least squares path modeling (PLS-PM) revealed the total effects under salt stress on the vermicomposting system.

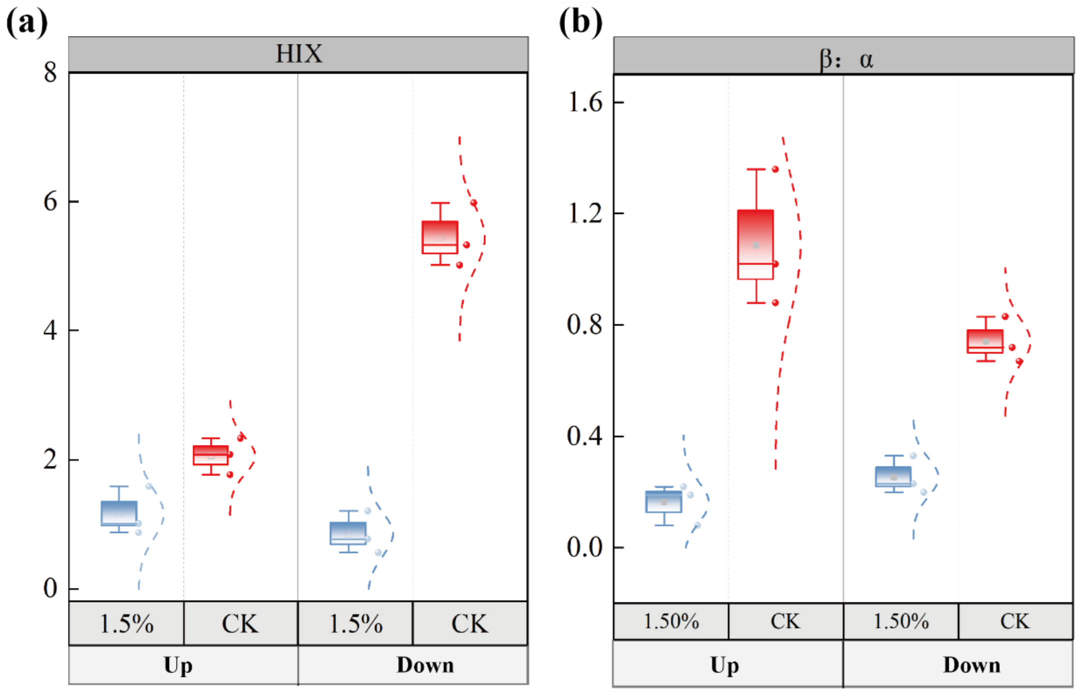

Fig.S1. Fluorescence characteristic parameters of the upper and lower layers of the substrate. (a) Humification index and (b) β:α index.

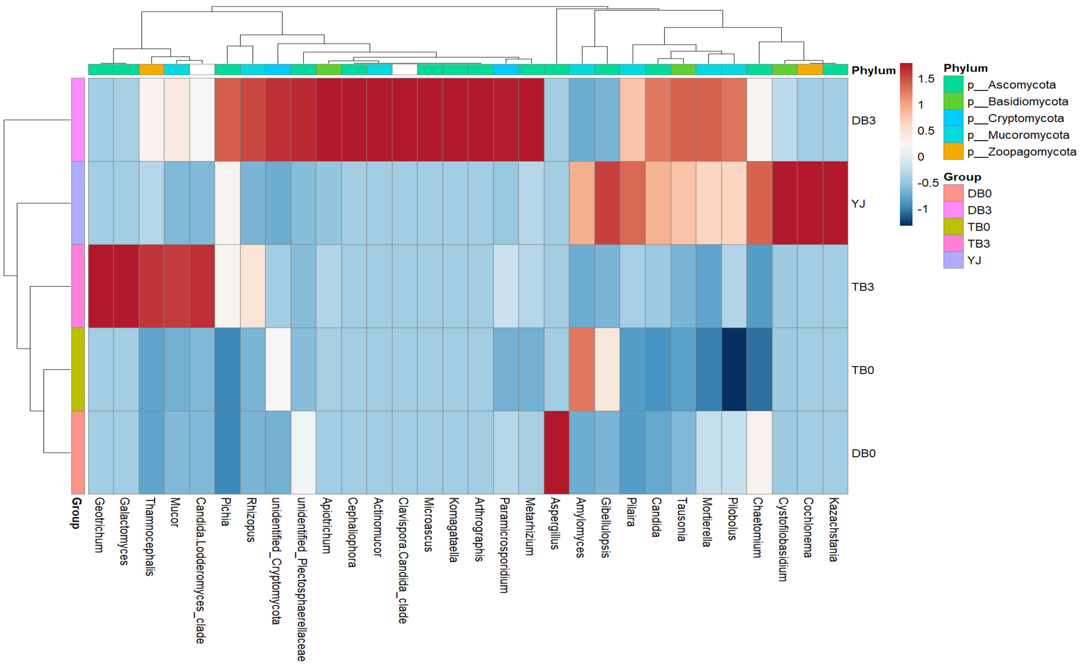

Fig.S2. The abundance of bacteria at the genus levels in the upper and lower layers of the substrate.

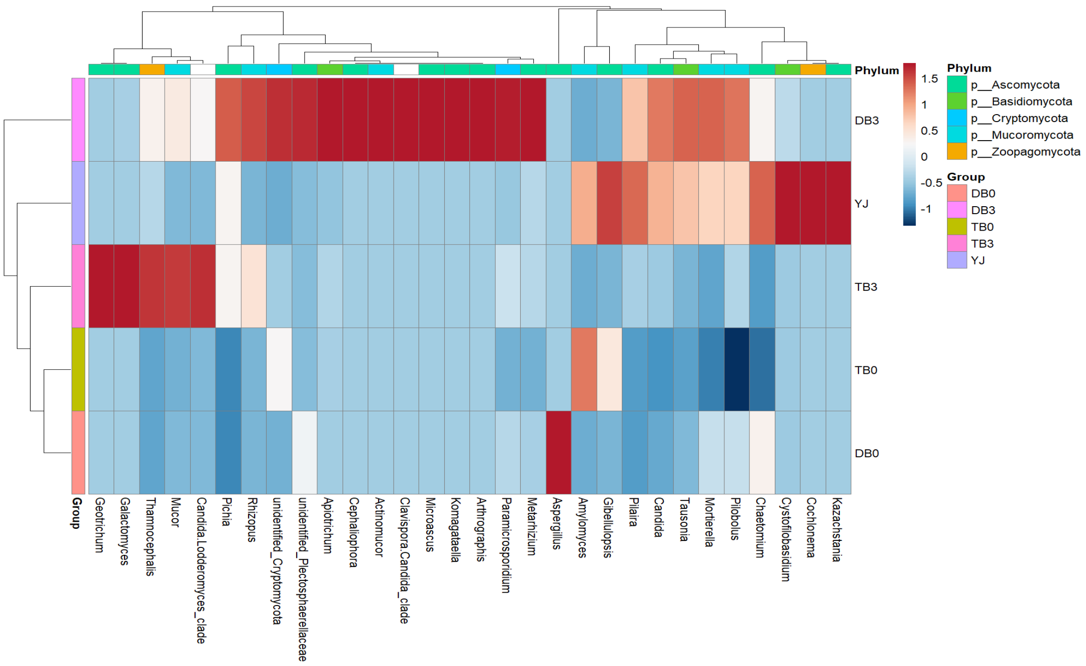

Fig.S3. The abundance of fungi genus levels in the upper and lower layers of the substrate.

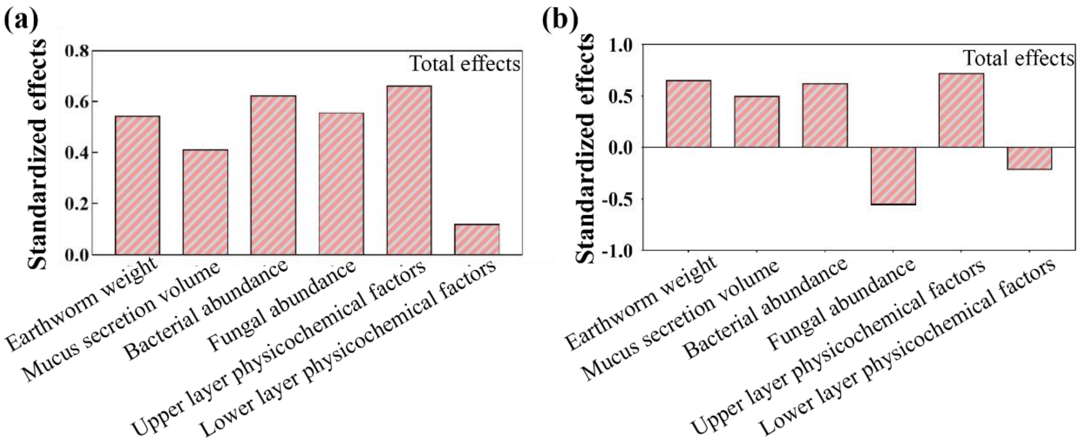

Fig.S4. Partial least squares path modeling (PLS-PM) revealed the total effects under salt stress on the vermicomposting system. (a) CK group and (b) salt group.
